# Osteoclastic bone resorption directly activates osteoblast function

**DOI:** 10.1186/ar3581

**Published:** 2012-02-09

**Authors:** Nobuyuki Udagawa

**Affiliations:** 1 Matsumoto Dental University, Shiojiri, Nagano, 399-0781, Japan

## 

Osteoclasts, the multinucleated cells that resorb bone, originate from cell cycle-arrested quiescent osteoclast precursors [1]. Mesenchymal osteoblastic cells are involved in osteoclast differentiation. Osteoclast precursors express RANK (a receptor of RANKL), recognize RANKL expressed by osteoblasts through cell-cell interaction and differentiate into osteoclasts in the presence of M-CSF. OPG, produced mainly by osteoblasts, is a soluble decoy receptor for RANKL. Deficiency of OPG in mice induces osteoporosis caused enhanced bone resorption. Elevated osteoblastic activity was suppressed by bisphosphonate administration in OPG-deficient mice. These results suggest that bone formation is accurately coupled with bone resorption. Collagen sponge disks containing BMP-2 were implanted into the dorsal muscle pouches in OPG-deficient mice. TRAP-positive osteoclasts and ALP-positive osteoblasts were observed in BMP-2-disks preceding the onset of calcification for one week. OPG and soluble RANK inhibited BMP-2-induced osteoclast formation but not the appearance of ALP-positive cells in OPG-deficient mice. We then examined how osteoblasts are involved in osteoclastogenesis other than RANKL expression, using RANKL-deficient mice. RANKL-deficient mice showed severe osteopetrosis due to loss of osteoclasts. Injection of RANKL into RANKL-deficient mice induced many osteoclasts in bone but not soft tissues [2]. These results suggest that osteoblasts determine the place of osteoclastogenesis from haemopoietic stem cells in bone. We next explored roles of osteoclasts in ectopic bone formation induced by BMP using op/op and c-fos-deficient osteopetrotic mice. The ectopic bones formed in op/op mice showed extremely rough surfaces, whereas those in wild-type mice showed smooth ones. Bone mineral density of BMP-induced ectopic bone in op/op mice was about 2-times higher than that in wild-type mice. TRAP-positive osteoclasts exhibit in outer of the ectopic bone in the wild-type mice. In op/op mice, although osteoclasts strongly exhibit in inside of the BMP-induced ectopic bone, TRAP-positive osteoclasts did not exhibit in outer of the BMP-induced ectopic bone. Furthermore, the accentuation of the BMP-induced ectopic bone formation did not exist in osteopetrotic c-Fos-deficient mice. In c-Fos-deficient mice, which are completely osteoclasts deficiency, the accentuation of the BMP-induced ectopic bone formation did not exist. Furthermore, there is no RANK-positive osteoclast progenitors in bone derived from c-Fos-deficient mice. These results suggest that RANK-positive osteoclast progenitors are positively regulate the signal of bone formation. In summary, osteoclastic bone resorption directly activates osteoblast function and osteoclasts are involved in normal bone morphogenesis.

## References

[B1] Mizoguchi T Identification of cell cycle-arrested quiescent osteoclast precursors in vivo J Cell Biol 2009 184 541 554 10.1083/jcb.200806139 19237598 PMC2654120

[B2] Yamamoto Y Osteoblasts provide a suitable microenvironment for the action of receptor activator of nuclear factor-kappaB ligand Endocrinology 2006 147 3366 3374 10.1210/en.2006-0216 16627581

